# Supplementary material for: Splice-Junction-Based Mapping of Alternative Isoforms in the Human Proteome
Source: Cell Rep. Author manuscript; Available in PMC 2020 Jan 15. (PMC6961840; doi:10.1016/j.celrep.2019.11.026)
Supplement: 2 [file NIHMS1546469-supplement-2.zip › DF1/PXD006675/PulmonaryArtery/PulmonaryArtery_7_SYPL1_ETSLHSPSNTSAPHSQGGIPPPTGL.pdf]

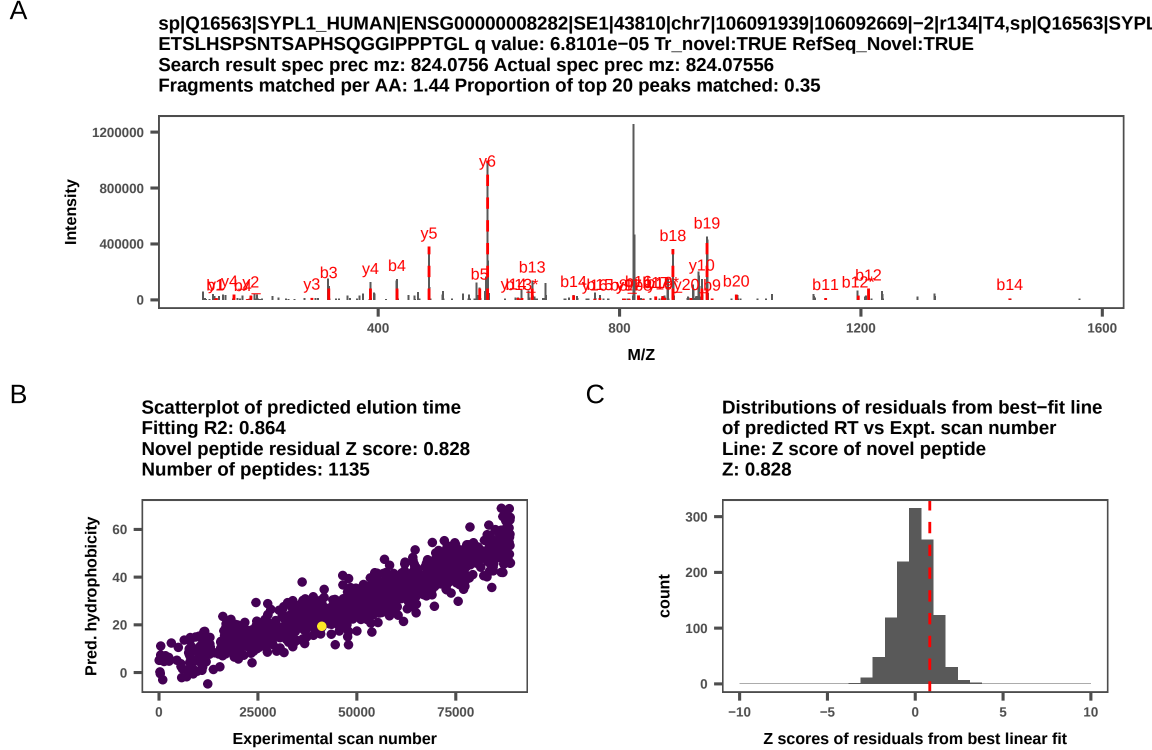
A
sp|Q16563|SYPL1_HUMAN|ENSG00000008282|SE1|43810|chr7|106091939|106092669|−2|r134|T4,sp|Q16563|SYPL
ETSLHSPSNTSAPHSQGGIPPPTGL q value: 6.8101e−05 Tr_novel:TRUE RefSeq_Novel:TRUE
Search result spec prec mz: 824.0756 Actual spec prec mz: 824.07556
Fragments matched per AA: 1.44 Proportion of top 20 peaks matched: 0.35
Intensity
1200000
800000
400000
0
y6
y5
b19
b18
y10
b3
y4
b4
b13
b5
y3
b20
b14
b11
b12
b14
400
800
1200
1600
M/Z
B
Scatterplot of predicted elution time
Fitting R2: 0.864
Novel peptide residual Z score: 0.828
Number of peptides: 1135
Pred. hydrophobicity
60
40
20
0
0
25000
50000
75000
Experimental scan number
C
Distributions of residuals from best−fit line
of predicted RT vs Expt. scan number
Line: Z score of novel peptide
Z: 0.828
count
300
200
100
0
−10
−5
0
5
10
Z scores of residuals from best linear fit
